# The who, what, and how of virtual participation in environmental research

**DOI:** 10.1007/s42532-023-00146-w

**Published:** 2023-03-08

**Authors:** Jennifer M. Holzer, Julia Baird, Gordon M. Hickey

**Affiliations:** 1 Environmental Sustainability Research Centre, Brock University, St. Catharines, Canada; 2 Department of Geography and Tourism Studies, Environmental Sustainability Research Centre, Brock University, St. Catharines, Canada; 3 Department of Natural Resource Sciences, McGill University, Montreal, Canada

**Keywords:** Stakeholder, Participation, Engagement, Workshop, Environmental governance

## Abstract

As a group of social scientists supporting a large, national, multi-site project dedicated to studying ecosystem services in natural resource production landscapes, we were tasked with co-hosting kick-off workshops at multiple locations. When, due to project design and the Covid-19 pandemic, we were forced to reshape our plans for these workshops and hold them online, we ended up changing our objectives. This redesign resulted in a new focus for our team—on the process of stakeholder and rightsholder engagement in environmental and sustainability research rather than the content of the workshops. Drawing on participant observation, surveys, and our professional experience, this perspective highlights lessons learned about organizing virtual stakeholder workshops to support landscape governance research and practice. We note that procedures followed for initiating stakeholder and rightsholder recruitment and engagement depend on the convenors’ goals, although when multiple research teams are involved, the goals need to be negotiated. Further, more important than the robustness of engagement strategies is flexibility, feasibility, managing expectations—and keeping things simple.

## Introduction

Stakeholder and rightsholder engagement (SRE) in action-oriented environmental research is challenging. The overarching challenge is building effective relationships among actors with different goals, needs, and abilities (Eaton et al. 2022). More specific challenges often lie in mismatches, like the requirements and pace of academic research and the timely needs for concrete, applied recommendations for practice (Mease et al. 2018, p 250; Holzer et al. 2018; Cash et al. 2006, p 8). This perspective article addresses the call for research to understand “barriers to engagement among researchers, practitioners, community stakeholders, and research and practice” (Eaton et al. 2022). As with many researchers, the Covid-19 pandemic presented additional roadblocks to the SRE aspects of our work, thwarting our ability to meet the original project objectives. We share some lessons we learned in pivoting from in-person to online stakeholder/rightsholder (SR) workshops due to unforeseen events, a situation that may be closer to the rule than the exception when undertaking SRE in research.

As a group of social scientists supporting a Canada-wide project to monitor, model, and improve governance of ecosystem services (the benefits nature provides to people), we coordinated kick-off workshops across six “working landscapes” (project-specified regions with protected areas, land and/or water “worked” by agriculture, extractive industries, etc., as well as residential areas). The primary objective of these workshops was to get key local actors in the same room, get acquainted, and, in the spirit of transdisciplinary research, begin the collaboration by building a shared understanding of the social-ecological system and discussing priority ecosystem services-related research in each landscape. Then, just two weeks prior to the first in-person workshop, Covid-19 restrictions canceled our plans and forced us to regroup. What could we, as researchers, our local co-host scientists, and the participants, get out of an online meeting? We ended up planning online workshops in three landscapes with an altogether different format and different objectives. This is the story of what we learned about the *who*, *what*, and *how* of doing effective SRE online. We hope sharing our experience is helpful to other researchers.

### The pivot from in-person to online workshops

Our original objective for the canceled in-person workshops had been a collaborative mapping exercise of each landscape’s social-ecological system, a challenging task meant to highlight the diverse perspectives of different actors, even about things individuals might take for granted—boundaries of the landscape, definitions of terms, priority places, or key ecosystem services. We deemed this task too demanding for an online workshop. Online workshop discussions have recognized advantages, such as saving time and costs and removing the need to travel, but also disadvantages—a bias toward those with good-quality internet access and familiarity with online meetings, and limits to social learning and building personal relationships (Nyugen et al. 2021, p 431–434; Shamsuddin et al. 2021, p 2–4; Lupton 2020). An online meeting of individuals who are already acquainted may also be quite different than convening a group online for the first time. With the stresses triggered by the pandemic, the workshop was deprioritized. Local researchers felt unsure if they should ask their contacts to participate in anything that would be perceived as demanding of their time.

After much discussion, our strategy became the following. To meet the project mandate to hold a kick-off workshop and the desire of local scientists to introduce their partners to the project and meet one another, we would facilitate a discussion about what ecosystem services were important to everyone in the room. To add a critical element, we asked participants whether they felt the ecosystem services concept was useful for fostering productive conversations. We hoped this would be a substantive hook for a meaningful conversation to build relationships, even virtually. But could these discussions possibly yield robust data? (Fell et al. 2020). We were doubtful. Like many researchers during the pandemic, we shifted our focus to process rather than content by necessity. However, the process of engagement—in this case, the question of who gets invited to participate in SR engagement—was embedded in our workshop planning from the very beginning. This then became our new research objective: to advance methods for SR selection in engagement, with an underlying motivation to increase diversity in participation and nudge local scientists to use systematic SR selection methods.

## Who gets invited and who attends?

*Researchers prioritize their formal partners, essential knowledge holders/decision makers, and special groups mandated by formal guidance.*

In three different landscapes, we looked to our local “landscape”^1^ researchers to suggest workshop invitation lists and encouraged them to consider the diversity of the invited group. Workshop invitations were then made by local researchers, in consultation with the authors. The focus on diversity and representation in workshop participation was motivated by our awareness of recommendations from recent literature (e.g., Bennett et al. 2021; Leventon et al. 2016; von der Porten & de Loë 2013; Prell et al. 2009; Reed et al. 2009) and the desire to “do right” at the start of a new project. We encouraged landscape researchers, for example, to reflect on the biases of their existing contacts, invite individuals beyond their existing contacts (Leventon et al. 2016, p 764), and list different actor groups and try to ensure that at least one individual from each group would be present at the meeting. We also shared what we would be asking them about their rationale for how invitations were extended to workshop participants and whether any measures were taken to consider diversity and representativeness in those invitations.^2^

### Recruitment favored formal partners and academic participants dominated

The three workshops each employed different participant recruitment strategies. In general, the landscape leads with whom we organized the workshops felt obligated, among other objectives, to use the opportunity to meet with researchers and partners who had joined the project during the proposal stage, since, for many, this would be the first opportunity they had to meet.

In addition to its core group of researchers, Workshop 1 invited only the four main non-academic project partners; these four individuals had never met before, so this was a chance for them to hear more about the project and to meet each other, to express what they might contribute to and get out of the project, as well as their perspectives on ecosystem services in their landscape. The exit survey showed that participants and organizers were very pleased with how deeply this small group was able to understand the project scope, discuss ecosystem services issues in their landscape, and get acquainted with one another.

Workshop 2 cast a wide net for participation, inviting all of their 40 + formal project partners; however, since many researchers and their students participated, academics dominated the participant group. Government decision makers and local producers were mostly absent. Several staff members from a formal project partner representing a confederation of Indigenous rightsholder groups attended the workshop.

Workshop 3 developed an invitation list that aimed to create a balance between academics, decision makers, resource users, and others, and to include participants in a related study for which participant interviews had recently been completed. Here, landscape-based scientists were particularly wary of bringing together individuals who might fall into conflict during discussions, and as a precaution, the workshop was carefully rehearsed. However, the resulting workshop was widely considered a success by both organizers and participants.

Prior to the workshop, we used an online survey to ask the workshop convenors which considerations influenced their process of inviting workshop participants. Each workshop was held online for between one and three hours. In each case, after a round of introductions, participants spent 10–15 min filling out a survey about the ecosystem services of greatest interest to them. The introduction to this activity acknowledged critiques of the ecosystem services concept (which may be particularly relevant for Indigenous participants, Pascua et al. 2017, p 472), but since ecosystem services was the focus of the project, the topic was still deemed appropriate as a focus of discussion. The meeting facilitator strived to create a supportive meeting environment and encourage everyone to share, whether in plenary or breakout groups. Plenary discussion focused on whether/how participants felt the ecosystem services concept could be useful for the project. After the workshops, we conducted follow-up surveys (online) with both workshop convenors and participants, respectively, about the quality of the workshop itself as well as their perceptions of the diversity and representativeness of the composition of the participant group. Workshop convenors reported on the logic they used to make workshop invitations, which differed in the three cases; all used a pragmatic approach, and none were based on a formal method (Table 1).

**Table 1:** Approaches for making workshop invitations by landscape lead scientists

Real-life methods for participant selection: Survey responses from local co-host scientists
“We invited only the partners that were already established through the ResNet proposal.” (Workshop 1, scientist)
“We settled on extending invitations to those already involved in [*redacted*], which is already a diverse group including key decision-makers, etc. (over 40 on the list). We worried that involving new voices for this short workshop would put new entrants at a disadvantage. We'd need more time than I was comfortable allotting for this workshop.” (Workshop 2, scientist)
“We first asked all landscape team members and diverse participants who have been involved in our ongoing research in the subject matter over the last three years. We then identified where there was less representation for certain perspectives out of those that had RSVP’ed for the workshop. We then used our research network to invite identify and invite additional participants from the underrepresented groups in our RSVP list (e.g., Indigenous leaders, producers, and agricultural industry representatives).” (Workshop 3, scientist)

### Approaching Indigenous rightsholders requires careful preparation

The inclusion of Indigenous rightsholders in these workshops was not mandatory, but desirable to the project. Workshop 2 included staff members from a non-profit organization that works on behalf of regional Indigenous groups. Workshop participants (who completed an anonymous survey after the workshops) commented that Indigenous voices should have been present or better represented at the workshops.

In this case, the structure of our project, with our work across multiple cases—mediated through landscape-based researchers—created barriers to forming direct relationships with Indigenous rightsholders (as well as stakeholders), making it challenging for us to follow current guidance for working with Indigenous rightsholders on environmental research, given that we had limited influence on invitations, workshop structure, etc. Early career researchers (such as the lead author) may feel especially challenged to build healthy relationships with local peoples and stakeholders due to the time and proximity required to develop such relationships (Filyushkina et al. 2022; Chapman et al. 2015, p. 337). We were fortunate to receive feedback from a community-engaged researcher with significant experience working with Indigenous communities about the need to limit requests from Indigenous groups for input and to focus more on the quality of engagement rather than the quantity. So, while straightforward, feasible approaches for researchers to plan participatory processes are desirable, our experiences underline the importance of sensitivity, careful planning, and a long-term perspective in including Indigenous rightsholders in scientific research, as outlined in the academic literature (e.g., Muller et al. 2019; Chilisa 2019; Bartlett et al. 2012) and in Canada’s guidelines for “Research Involving First Nations, Inuit and Métis Peoples of Canada” (Canadian Institutes of Health Research, Natural Sciences and Engineering Research Council of Canada, & Social Sciences and Humanities Research Council of Canada 2018).

## What is the quality of virtual engagement?

*The quality of interactions can be highly rewarding to a variety of actors, as long as objectives and expectations are adjusted and primed appropriately*.

### Convenors and participants valued the opportunity to meet remotely

Participants appreciated the opportunity to meet and engage with one another. When asked about the quality of the meeting in the survey, responses were overwhelmingly positive, although some acknowledged that they less prefer virtual meetings. Participants expressed appreciation for the opportunity to meet others concerned about similar issues (“Fantastic to have the workshop like this”; “The discussions were energizing…”), while also recognizing shortcomings of having invitation-only, online workshop (e.g., “They [participants] were likely better informed than the average”). When asked about the representativeness of participants vis-a-vis local ecosystem services issues, participants named sectors and individuals they were missing from the conversation (e.g., “individual landowners and users,” “other provincial departments,” and “I would be interested in hearing more from an Indigenous background, agricultural background, instead of just the scientist/academic background.”).

Likewise, the place-based scientists in all three cases expressed relief that they were able to convene their array of project partners and were pleased with the results of the meeting. While it seemed (from participant observation) that the fact that the meeting was a funding requirement was crucial in making the workshops a reality, it also seemed (from landscape leads’ feedback) that they felt that the meeting was worthwhile and beneficial for relationships with their landscape SR.

### Data collection opportunities were constrained by the format

Changes in our research design were pragmatic; they served to meet our mandate to hold workshops in the first year of the five-year project (with leniency granted regarding timing due to the Covid-19 pandemic). As others have shown, it was harder to reach stakeholders, (non-academic) stakeholder priorities shifted away from scientific research, and stakeholders had less time to spend on participation (Kopsel et al. 2021, p 197). We were concerned about getting participants to attend and observed that the online format placed an additional barrier to the diversity of workshop participants. For example, certain SRs would have preferred in-person workshops and this likely affected whether they attended (or attended all of) a remote meeting. To be sensitive toward participants and to allay fears of local scientists that any controversial question might spark conflict, we endeavored to create an agenda that was substantive but focused on fundamental questions.

### Tensions between priorities of scientist teams

There were several implications of the fact that workshops were co-developed and co-facilitated by landscape leads together with the cross-cutting research team (i.e., the authors). The most important was that we were entering into an existing set of relationships and projects already underway; an example of this was that landscape leads controlled the workshop invitations. This was a constraint that limited our ability to collect data that was representative of diverse interests in landscape governance. On the other hand, because landscape leads controlled the workshop invitations, they could encourage attendance to promote strategic relationship-building. Certain elements unfolded throughout the workshop planning process that required negotiation among the two teams, and these types of dilemmas are a likely reality for many place-based research projects, especially those with multiple case studies and/or research groups.

## How should researchers plan for virtual engagement?

### Strive for minimalism, pragmatism, and simplicity

As described above, workshop objectives were scaled down to meet the mandated deliverable (i.e., three workshops in each of six landscapes over a five-year period) and the need to figure out how to design a meeting format that would be perceived as successful to all involved parties. At the outset of workshop planning, we had advocated for systematic and equitable SR recruitment strategies, aiming to make meetings representative of the full range of SR diversity in the landscape. However, in planning, facilitating, and reviewing feedback from the workshops, we saw that a main difficulty is keeping things simple, requiring a feasible and accessible strategy for SRE in research.

### Criteria for participant recruitment should be outlined in the project proposal

While it has become common in conservation, natural resource management, and related fields for project funding to require participatory workshops, many scientists have no designated funding or training for conducting SR engagement (Arnott et al. 2020, p 40). This may not allow scientists to benefit from the evidence and experience gained through engaged research on ecosystem governance. It would be ideal for projects to outline objectives and guidelines for SRE early, even at the proposal stage, as it has been shown that the choice of research approach strongly depends on funding requirements (Jahn et al. 2021, p 343; Holzer et al. 2018). The following questions might be considered:

- What type of workshop will we hold and what will its objectives be? (see, e.g., Ørngreen & Levinsen 2017, p 72–74);
- By what criteria should the appropriateness of the makeup of a participant group be judged? (see, e.g., Ferguson et al. 2022; Leventon et al. 2016, p 765–767; Reed et al. 2009, p S13-S14);
- How will organizers balance appropriate diversity and representation without increasing group size to an unwieldy number?;
- How will the success of the activity be gauged? (See guidelines for evaluation of transdisciplinary projects, see, e.g., Plummer et al. 2022, p 958; Holzer et al. 2018; Reed et al. 2018, S11-S13; van Drooge & Spaapen 2017, p 752–754).

### Researcher flexibility can foster relationship-building

In a situation rife with stress and surprise, such as the Covid-19 pandemic, it may not be possible to follow through on objectives envisioned during a different status quo. We were not able to collect robust data—despite a complete change of plan. We did, however, meet an underlying objective of relationship-building, making an investment in this stakeholder community, in this research project, and for potential unknown benefits in the future.

Adjustments made during the scientific process can result in unanticipated benefits; forced to hold workshops online, we changed our expectations for what workshops would deliver but held onto the goal of getting participants acquainted with the project and each other by facilitating substantive conversations. Reflecting on workshop planning, execution, and feedback, we realize that the process helped to draw out the values and priorities of the organizing scientists and bring them into conversation with one another; this itself is beneficial, especially at the beginning of a transdisciplinary project.

## Conclusion

Virtual workshops in the context of environmental research can help stakeholder and rightsholder participants share views and discuss governance challenges, thereby supporting the core work of collaboration in the environmental research-practice space: collectively defining the problems to be addressed and negotiating pathways toward sustainability (Cockburn et al. 2018, p 13–14). Workshops, particularly online workshops, will never replace the need for ongoing relationship-building, community engagement, reflexivity, or adaptive governance. SR participation should be considered an essential part of the scientific process, and, therefore, be written into proposals, grant agreements, and budgets as a matter of course. A related key issue is gaining buy-in and cooperation from the collaborators needed to access and organize SR as early as possible in the process. Crucially, willing scientists from diverse fields—not just social scientists—need to see relationship-building and reflexivity work as an integral part of conducting applied environmental science and be trained and supported to do so. Further work to create accessible, feasible guidelines to support scientists in incorporating SRE in their work, and particularly doing so online, remains necessary. There is also a need to further develop SR engagement training resources for scientists and funders from diverse backgrounds.

## Footnotes

1 The definition of the three working landscapes that participated in the virtual workshops were defined by the grant that funded the project; each of these landscapes had a history of prior research and engagement with regional and local actors (academic, government, non-profit, and others).

2 While we had hoped that planning SR engagement would be systematic, this was not on the level of formal procedures for balanced participation, such as is mandated for Extension programs in the United States. In that context, civil rights goals for extension programs interpret ‘balanced participation’ to mean that the gender and race of individuals served by the program is within 20% of the local demographic (according to the census or local records) (e.g., NC Cooperative Extension Civil Rights Plan, https://evaluation.ces.ncsu.edu/nccepolicies/).
